# Non-pharmacological therapies for treating non-motor symptoms in patients with Parkinson’s disease: a systematic review and meta-analysis

**DOI:** 10.3389/fnagi.2024.1363115

**Published:** 2024-04-26

**Authors:** Yu Zhang, Shuang Liu, Ke Xu, Yan Zhou, Yiwei Shen, Zhengnan Liu, Yan Bai, Shun Wang

**Affiliations:** ^1^School of Acupuncture-Moxibustion and Tuina, Heilongjiang University of Traditional Chinese Medicine, Harbin, China; ^2^Institute of Acupuncture and Moxibustion, Heilongjiang Academy of Traditional Chinese Medicine, Harbin, China

**Keywords:** non-drug therapy, Parkinson’s disease, acupuncture, cognitive behavioral therapy, exercise, repetitive transcranial magnetic stimulation, non-motor symptoms, meta-analysis

## Abstract

**Objective:**

The non-motor symptoms of Parkinson’s disease (PD) are an important part of PD. In recent years, more and more non-drug interventions have been applied to alleviate the non-motor symptoms of PD, but the relevant evidence is limited. This systematic review and meta-analysis was designed to evaluate the efficacy of non-drug interventions in patients with non-motor symptoms in patients with PD.

**Methods:**

Seven databases, including Pubmed, Embease, Cochrane Library, China National Knowledge Infrastructure (CNKI), Wanfang database (WANFANG), VIP database (VIP), and China Biomedical Literature Service System (CBM) were searched from the establishment of the database to December 2023. Non-drug interventions such as acupuncture, cognitive behavioral therapy (CBT), exercise, repetitive transcranial magnetic stimulation (rTMS), and non-motor symptoms of Parkinson’s disease were selected as search words, and two independent evaluators evaluated the included literature’s bias risk and data extraction. The therapeutic efficacy was evaluated by the Parkinson’s Disease Sleep Scale (PDSS), Hamilton Depression Scale (HAMD), Beck Depression Inventory (BDI), Hamilton Anxiety Scale (HAMA), Montreal Cognitive Assessment (MoCA), Minimum Mental State Examination (MMSE), and Parkinson’s Disease Questionnaire-39 (PDQ-39). RevMan 5.4.1 (Reviewer Manager Software 5.4.1). Cochrane Collaboration, Oxford, United Kingdom analyzed the data and estimated the average effect and the 95% confidence interval (CI). A heterogeneity test is used to assess differences in the efficacy of different non-drug treatments.

**Results:**

We selected 36 from 4,027 articles to participate in this meta-analysis, involving 2,158 participants. Our combined results show that: PDSS: [mean difference (MD) = −19.35, 95% CI (−30.4 to −8.28), *p* < 0.0006]; HAMD: [MD = −2.98, 95% CI (−4.29 to −1.67), *p* < 0.00001]; BDI: [MD = −2.69, 95% CI (−4.24 to 4.80), *p* = 0.006]; HAMA: [MD = -2.00, 95% CI (−2.83 to −1.17), *p* < 0.00001]; MMSE: [MD = 1.20, 95% CI (0.71 to 1.68), *p* < 0.00001]; CoMA: [MD = 2.10, 95% CI (−0.97 to 3.23), *p* = 0.0003]; PDQ-39: [MD = −4.03, 95% CI (−5.96 to −1.57), *p* < 0.00001].

**Conclusion:**

The four non-drug measures used in our review showed significant improvements in sleep, depression, anxiety, cognition, constipation, and quality of life compared with the control group, and no serious adverse events were reported in the included research evidence, and we found that there were some differences among the subgroups of different intervention methods, but due to the less literature included in the subgroup, and the comparison was more indirect. So, we should interpret these results carefully.

**Systematic review registration:**

www.crd.york.ac.uk/PROSPERO, identifier CRD42023486897.

## Introduction

Parkinson’s disease (PD), first described in 1817, has developed into the second most common neurodegenerative disorder (Jankovic, 2008). The onset age of the disease is mainly between 65 and 70 years old, and the prevalence rate increases with age. Studies have shown that the prevalence and incidence of Parkinson’s disease will increase by more than 30% by 2030, which will lead to more and more elderly who have Parkinson’s disease, increasing the burden and cost to society and families (Stoker and Greenland, 2018). Genetic studies of PD show that a mutation in a pathogenic gene can only explain a small part of the cause of PD, while the etiology of most sporadic cases is still unknown (Tysnes and Storstein, 2017). The pathological feature of PD is the degeneration and loss of dopaminergic neurons in the substantia nigra, which will lead to damage to the basal ganglion and its nerve loop, which will directly affect motor function regulation in patients with PD (Assogna et al., 2020). The diagnosis of PD mainly depends on clinical symptoms such as rest tremors, bradykinesia, muscle rigidity, abnormal posture, and gait instability, etc. In addition, drugs for Parkinson’s disease treatment primarily focus on typical motor symptoms, such as increasing dopamine levels and stimulating dopamine receptors, or offering symptomatic relief with medications like levodopa, amantadine (Marsili et al., 2018). These medications often require long-term use. However, the decline resulting from prolonged treatment will indirectly increase the likelihood of non-motor symptoms in individuals with PD and add to the burden on caregivers. It has been reported that non-motor symptoms in patients with PD usually precede motor symptoms for years or even decades and may progress with the deterioration of motor symptoms (Sveinbjornsdottir, 2016). Common non-motor symptoms of PD include hyposmia, color vision deficiency, hallucinations, pain, anxiety, cognitive dysfunction, dementia, sleep disturbance, and bladder hyperreflexia (Lotankar et al., 2017). In our review, cognitive impairment, sleep status, depression and anxiety, and the quality of life of patients with PD were reported. Other studies have shown that 20% of PD patients will show obvious non-motor clinical symptoms; although the current research continues to deepen the understanding of non-motor symptoms, the timely diagnosis is still lagging. Moreover, finding appropriate treatment methods is still the biggest potential obstacle (Schapira et al., 2017). At present, non-motor symptoms are mostly treated with symptomatic drugs, such as antidepressant drugs and anti-anxiety drugs, but long-term use of such drugs will produce a certain degree of drug resistance. The progression of non-motor symptoms will be further reflected in the impact on the quality of life of patients (Assogna et al., 2020).

In our review, acupuncture, cognitive behavioral therapy, exercise and repetitive transcranial magnetic stimulation were selected as the main non-drug interventions. Clinical studies on non-motor symptoms of PD were screened to compare and evaluate the efficacy of a variety of non-drug methods, in order to provide evidence-based basis for clinical practice to choose the best non-drug treatment. Acupuncture, an important means of treatment in Chinese traditional medicine, is an important part of complementary and alternative therapy. A large number of studies have been carried out on the application of acupuncture in the treatment of motor dysfunction caused by central and peripheral neuropathy (Liu et al., 2017; Yang et al., 2023a,b). In addition, it has been reported that acupuncture can affect the corresponding neurotransmitters and endogenous substances by stimulating special acupoints of the body, and can act on targeted neurons and synaptic remodeling or immune response to relieve symptoms such as insomnia and depression (Yin et al., 2017). In the current clinical research, acupuncture has achieved considerable effect in improving motor symptoms and non-motor symptoms of PD, and many positive results of related potential mechanisms have been reported in acupuncture intervention PD animal model (Zeng and Zhao, 2016; Yu et al., 2020).

Cognitive behavioral therapy (CBT) is a psychosocial intervention designed to improve mental health and regulate emotions. It is a short, problem-oriented approach that helps patients identify and correct dysfunctional thoughts, assumptions, and behavioral patterns (Hofmann et al., 2012). In one meta-analysis, CBT had a large effect on depression (Yarwood et al., 2023), while in another randomized controlled trial, the CBT group reduced depressive or anxiety symptoms, although it lacked large-scale clinical controlled trials, but showed great potential (Carney et al., 2017).

In our review, Chinese traditional exercise methods such as Tai Chi, Qigong and Western exercises such as yoga are included in exercise therapy. This kind of exercise combines balance, flexibility and neuromuscular coordination with cognitive activities to improve the motor and non-motor symptoms of patients with PD by improving physical awareness, concentration, imagery, multitasking and planning and goal-oriented training (Deuel and Seeberger, 2020). With the intervention of exercise therapy, the improvement of motor symptoms (such as balance, gait) and non-motor symptoms (cognition, depression) of PD patients can significantly improve their quality of life (Song et al., 2017; Tang et al., 2019).

Repetitive transcranial magnetic stimulation (rTMS), a non-invasive, highly tolerant treatment, mainly stimulates nerve tissue and regulates nerve activity by inducing magnetic field to generate spatially distributed current through the surrounding medium. The evidence-based guidelines used in the treatment of rTMS show that rTMS is considered to be a significant intervention for PD, motor symptoms of stroke, cognitive impairment of Alzheimer’s disease, anxiety and depression and other mental symptoms (Lefaucheur et al., 2020). The efficacy of fluoxetine and rTMS was compared in a randomized, blind, clinical trial. The results show that rTMS can be safely used in the treatment of depressive symptoms in patients with PD (Boggio et al., 2005).

## Methods

This systematic review and meta-analysis was performed according to Preferred Reporting Items for Systematic Reviews and Meta-Analyses (PRISMA) guidelines (Moher et al., 2009).

### Literature search and selection

We have systematically searched the literature from the establishment of the database to December 2023 in English and Chinese, including seven databases: 3 English databases PubMed, Embase, and Cochrane Central Register of Controlled Trials and 4 Chinese databases: China National Knowledge Infrastructure (CNKI), Wanfang database (WANFANG), VIP database (VIP) and Chinese Biomedical Literature Service system (CBM). Search strategies are mainly composed of diseases (“Parkinson’s disease,” “Parkinsonism”), interventions (“Non-pharmacological therapies,” “non-drug therapies,” “acupuncture,” “repetitive transcranial magnetic stimulation,” “cognitive therapy,” “cognitive behavior therapy,” “exercise therapy,” “exercise therapy,” “Tai Ji,” “Qigong,” “dance”) and research types (“randomized controlled trial”). “RCT” consists of three parts. Medicine terms determine the subject words by searching MeSH and Emtree and using the combination of topic words and free words to form the final search. See Appendix 1 for the specific search. In addition, we also searched the meta-analysis related to this topic and downloaded and read the reference literature to get all the information.

### Eligible criteria

We searched only RCT studies published in peer-reviewed journals, and the inclusion criteria of this study were determined according to the PICOS principles.

P (participants): participants met the clinical criteria for PD with obvious non-motor symptoms such as sleep disorders, cognitive impairment, mental disorders including anxiety and depression, autonomic symptoms including constipation, etc. the diagnostic criteria of PD can be referred to the internationally recognized consensus criteria such as the British Parkinson’s Society Brain Bank criteria (Rajput, 1993) or the International Movement Disorder Society (MDS) versions of the Unified Parkinson’s published by MDS. The Disease rating Scale (Movement Disorder Society Task Force on Rating Scales for Parkinson’s Disease, 2003) and the Chinese diagnostic criteria for Parkinson’s disease (Parkinson’s Disease and Movement Disorders Group, Chinese Society of Neurology, Parkinson’s Disease and Movement Disorders, Neurology Branch of Chinese Medical Doctor Association, 2016). The age of the participants is more than 18, but their sex, race and course of disease are not restricted.

### Exclusion criteria

1. Duplicate literature.
2. Non RCT, animal experiment, meta-analysis, review, conference and dissertation.
3. The pre-intervention method of the experimental group was more than two kinds of composite intervention methods.
4. The study participants were combined with other severe diseases, such as stroke, Alzheimer’s disease, and other diseases that share symptoms with PD.
5. The outcome indicators did not meet the needs of this study and the literature data were incomplete or could not be obtained complete data.

### Outcome measures

O (outcome): select the corresponding evaluation method for the non-motor symptoms of the participants. Hamilton Depression Scale (HAMD) and Beck Depression Inventory (BDI) were used to evaluate the depression of participants. Hamilton Anxiety Scale (HAMA) was used to evaluate anxiety symptoms. The Montreal Cognitive Assessment (MoCA) and Minimum Mental State Examination (MMSE) assessed participants’ cognitive function. Parkinson’s Disease Sleep Scale (PDSS) was used to evaluate participants’ sleep problems. The secondary outcome measures included the Parkinson’s Disease Questionnaire-39 (PDQ-39).

### Literature screening and data extraction

Two evaluators (YuZ and SL) use Endnote 20 to screen and review the literature, conduct a preliminary literature screening through the title and abstract, and then obtain the full text of the qualified papers for secondary screening. If the literature data is missing, contact the author through e-mail or telephone to obtain complete data, and finally, determine the inclusion of the literature and extract the relevant data included in the literature. It includes research characteristics such as study author, publication year, research source, clinical characteristics such as age, sex, number of cases, course of disease, intervention mode of experimental group/control group, and outcome index. Study screening and data extraction results were cross-checked, and any discrepancies were resolved through discussion with a third author (SW). Each study’s primary and secondary outcomes were extracted as mean and standard deviation (mean ± SD). If the study measured the outcome data at different time points, the data immediately after the intervention were used.

### Bias risk assessment

The two evaluators (YuZ and SL) used the Cochrane Collaboration Network bias risk assessment tool version 5.0.2 to evaluate the quality of the methodology included in the study. The risk tool includes seven important sources of bias (selective bias, implementation bias, measurement bias, follow-up bias, reporting bias, and other biases). It is described as “low risk,” “high risk,” and “risk ambiguity.” If any differences arise, the third evaluator (SW) will negotiate and resolve them. When any scale had more than 10 eligible studies, funnel plot was used to detect publication bias.

### Statistical analysis

We compared the results of similar non-drug interventions and conducted a meta-analysis using RevMan5.4.1 (Reviewer Manager Software 5.4.1; Cochrane Collaboration, Oxford, United Kingdom). For the data of the study’s results as continuous variables, the mean ± standard deviation or standardized mean deviation and 95% confidence interval (CI) were used for data analysis, and *I*^2^ statistics and *p*-values were used to evaluate heterogeneity. If *I*^2^ > 50% or *p* < 0.1, it is considered that there is substantial heterogeneity among the studies, and if *I*^2^ > 75%, there is significant heterogeneity. When there is significant heterogeneity, we will find the source of heterogeneity through subgroup analysis and sensitivity analysis. Subgroup analysis and sensitivity analysis were carried out according to the mode of intervention. If *I*^2^ > 50%, use the random effect model; otherwise, use the fixed effect model to analyze the data. If the number of studies included is minimal or the data is unsuitable for quantitative synthesis, the results are descriptively analyzed.

## Results

**Figure 1:**
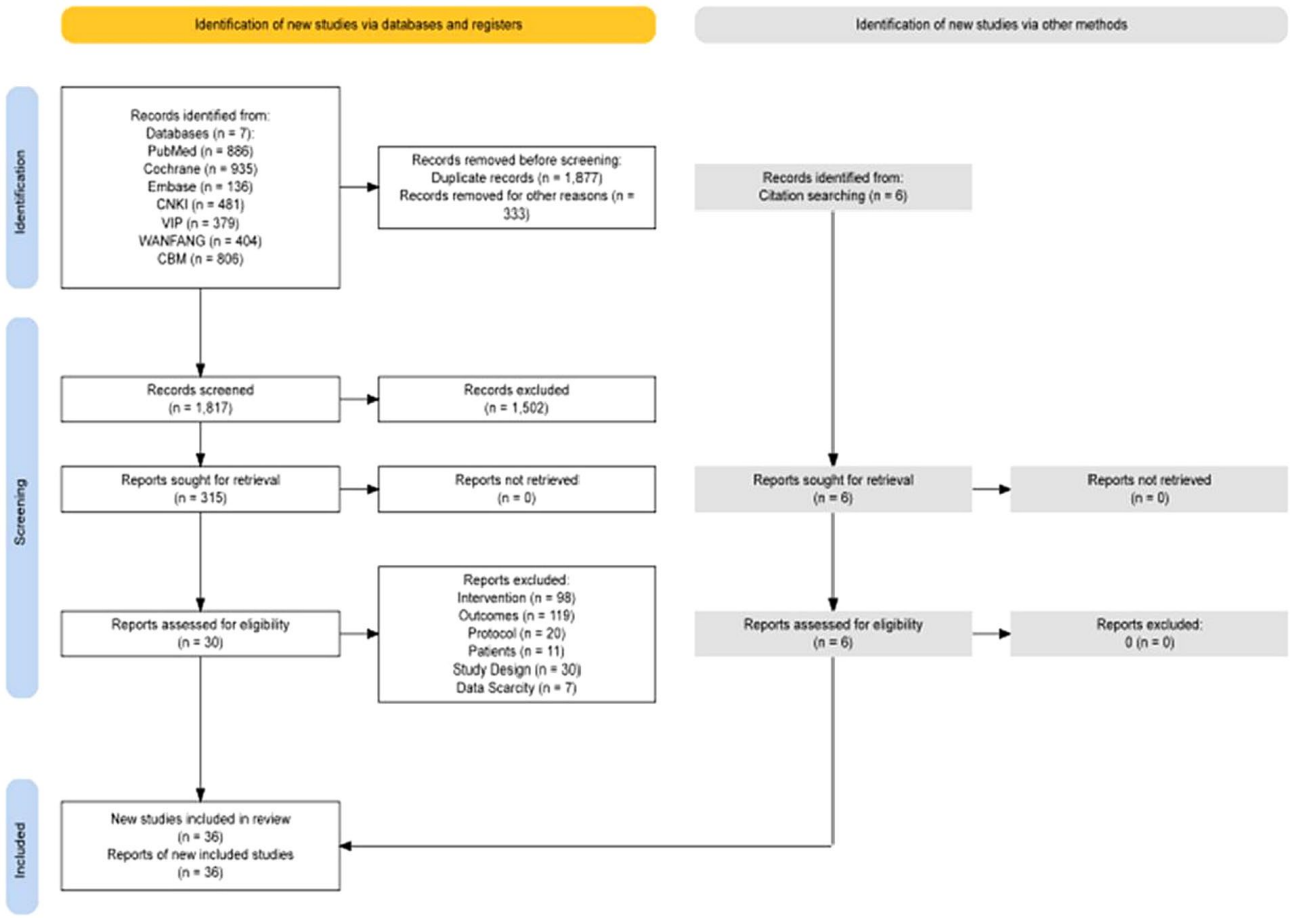
Flow chart of study selection.

According to the retrieval strategy, 4,027 articles, 1,957 in English and 2,070 in Chinese, were retrieved from 7 databases, and the full text was further searched. After screening according to the inclusion and exclusion criteria, 36 articles were included in the systematic review. Thirty-five articles were analyzed quantitatively, and one was systematically evaluated. See Figure 1 for details. There were 23 English articles, 13 Chinese articles, and randomized controlled trial (RCT) = 36, involving 2,158 patients. Non-drug interventions include acupuncture = 8 (Xia et al., 2012; Kluger et al., 2016; Kong et al., 2017; Zhang et al., 2018, 2020; Fan et al., 2022; Han et al., 2022), CBT = 11 (Veazey et al., 2009; Dobkin et al., 2011; París et al., 2011; Petrelli et al., 2014; Bernini et al., 2019; Rodgers et al., 2019; Dobkin et al., 2020; Moonen et al., 2021; Fan et al., 2022; Yang et al., 2023a,b), exercise = 10 (Rios Romenets et al., 2015; Ferreira et al., 2018; Wu et al., 2018; Kalyani et al., 2019; Solla et al., 2019; Moon et al., 2020; Zhu et al., 2020; Cao et al., 2021; Wang et al., 2022; Xi and Bi, 2022), rTMS = 7 (Cardoso et al., 2008; Su et al., 2012; Wu et al., 2019; Li et al., 2020; He et al., 2021; Chen et al., 2022; Cheng et al., 2022), the intervention of the control group mainly included sham stimulation or placebo, waitlist, routine treatment of treatment as usual (TAU), such as Clinical Monitoring, standard physical rehabilitation, antiparkinson drugs, etc. The sources of literature are mainly concentrated in Asia (Su et al., 2012; Xia et al., 2012; Zhou et al., 2014; Kong et al., 2017; Pan, 2018; Wu et al., 2018; Zhang et al., 2018; Wu et al., 2019; Li et al., 2020; Zhang et al., 2020; Zhu et al., 2020; Cao et al., 2021; He et al., 2021; Chen et al., 2022; Cheng et al., 2022; Fan et al., 2022; Han et al., 2022; Lu et al., 2022; Wang et al., 2022; Xi and Bi, 2022; Yang et al., 2023a,b), North America (Veazey et al., 2009; Dobkin et al., 2011; Rios Romenets et al., 2015; Kluger et al., 2016; Dobkin et al., 2020; Moon et al., 2020), Europe (París et al., 2011; Petrelli et al., 2014; Bernini et al., 2019; Solla et al., 2019), Oceania (Kalyani et al., 2019; Rodgers et al., 2019; Moonen et al., 2021), South America (Cardoso et al., 2008; Ferreira et al., 2018). See Table 1 for details.

**Table 1:** Characteristics of the included clinical trials involving non-pharmacological therapies for Parkinson’s disease.

Characteristics of literature			Characteristics of the participants						
References	Years	Country	Age (y)	Sex (M/F)	Sample	Disease duration (y) /H&Y stage	Drug use/LEDD (mg/day)	Intervention	Measured outcomes
Kong et al.	2017	Singapore	C: 62.9 ± 9.7 E: 66.4 ± 6.5	C: 13/7 E: 14/7	C: 20 E: 20	C: 50.1 ± 26.4 E: 87.2 ± 53.2(months)/NR	NR/ C: 615.2 ± 347.9 E: 637.8 ± 394.3	C: Sham AT E: AT	(1) PDQ-39
Kluger et al.	2016	United States	C: 63.0 ± 13.0 E: 64.4 ± 10.3	C: 30/17 E: 29/18	C: 47 E: 47	NR/HYI-IV	NR/ C: 628.6 ± 482.9 E: 558.9 ± 379.3	C: Sham AT E: AT	(1) PDSS (2) PDQ-39
Han et al.	2022	China	C: 61 ± 3 E: 61 ± 3	C: 28/20 E: 30/18	C: 48 E: 48	C: 4.4 ± 2.6 E: 4.1 ± 2.8/HYI-III	Madopar/ C: 375 to 750 E: 375 to 750	C: AD E: AD+AT	(1) PDQ-39
Zhang et al.	2018	China	NR	NR	C: 30 E: 30	NR	NR	C: EAT E: AT	(1) BSS (2) CCS
Lu et al.	2022	China	C: 67.36 ± 8.83 E: 67.81 ± 7.27	C: 18/15 E: 16/16	C: 33 E: 32	C: 36.03 ± 18.78 E: 37.22 ± 22.24 (Mouths)/HYI-III	Madopar/ C: 200 D: 200	C: AD E: AD+AT	(1) HAMD
Zhang et al.	2020	China	C: 55.32 ± 3.02 E: 52.16 ± 3.56	C: 26/22 E: 21/27	C: 48 E: 48	C: 4.12 ± 1.21 E: 4.46 ± 1.32/NR	NR	C: AD E: AD+AT	(1) HAMD (2) PDSS
Xia et al.	2012	China	C: 72 ± 8 E: 72 ± 7	C: 20/10 E: 22/8	C: 30 E: 30	C: 5.8 ± 4.0 E: 6.7 ± 3.0/NR	Madopar C: 600 E: 600	C: TAU E: TAU+EAT	(1) HAMD
Fan et al.	2022	China	C: 62.66 ± 6.94 E: 61.03 ± 9.80	C: 15/17 E: 19/13	C: 32 E: 32	C: 4.00 (2.00–8.00) E: 5.00 (4.00–9.00)/HYI-IV	NR	C: Sham AT E: AT	(1) HAMA (2) PDQ-39
Moonen et al.	2021	Netherlands	C: 63.3 ± 8.4 E: 63.3 ± 7.2	C: 12/12 E: 11/13	C: 24 E: 24	C: 4.7 ± 4.0 E: 7.4 ± 5.6/HYI-III	NR/ C: 760.5 ± 517.7 E: 839.7 ± 1018.6	C: CM E: CBT	(1) HAMA (2) HAMD
París et al.	2011	Spain	C: 65.42 ± 9.60 E: 64.75 ± 9.19	C: 7/5 E: 7/9	C: 12 E: 16	C: 8.25 ± 9.22 E: 6.94 ± 4.58/HYI-III	NR	C: SH E: CBT	(1) PDQ-39 (2) MMSE
Petrelli et al.	2014	Germany	C: 69.2 ± 4.9 E: 69.1 ± 11.6	C1: 9/12 C2: 7/15 E: 12/10	C1: 21 C2: 22 E: 22	C1: 5.41 ± 4.4 C2: 5.94 ± 4.68 E: 5.51 ± 3.29/NR	NR	C1: No treatment C2: U-CBT E: S-CBT	(1) MMSE (2) PDQ-39
Dobkin et al.	2019	United States	C: 64.80 ± 9.62 E: 65.62 ± 9.76	C: 18/17 E: 17/20	C: 35 E: 37	C: 5.65 ± 4.20 E: 6.95 ± 7.82/NR	NR	C: TAU E: T-CBT + TAU	(1) HAMD (2) BDI (3) HAMA
Zhou et al.	2014	China	T: 60.05 ± 2.06	T: 46/64	C: 50 E: 50	NR	NR	C: venlafaxine E: venlafaxine + CBT	(1) HAMD
Veazey et al.	2009	United States	T: 72	T: 10	C: 5 E: 5	NR	NR	C: TS E: CBT	(1) MMSE (2) PDQ-39
Dobkin et al.	2011	United States	C: 65.44 ± 11.23 E: 63.73 ± 9.89	C: 23/16 E: 25/16	C: 39 E: 41	C: 6.13 ± 5.56 E: 6.53 ± 5.53/HYI-III	NR	C: CM E: CBT + CM	(1) HAMD (2) BDI (3) HAMA
Bernini et al.	2019	Italy	C: 69.33 ± 7.72 E: 71.18 ± 7.04	C: 11/7 E: 6/11	C: 18 E: 17	C: 10.67 ± 7.36 E: 7.18 ± 3.19/HY ≤ IV	NR	C: SPR E: SPR + CBT	(1) MMSE (2) MoCA
Yang et al.	2023	China	C: 61.42 ± 9.72 E: 63.06 ± 7.97	C: 16/17 E: 15/18	C: 33 E: 33	C: 4.30 ± 1.56 E: 4.33 ± 1.50/HY < III	NR	C: TAU E: TAU + CBT	(1) HAMD (2) HAMA
Pan et al.	2018	China	C: 44.5 ± 5.4 E: 43.7 ± 5.1	C: 17/17 E: 19/15	C: 34 E: 34	C: 6.9 ± 5.2 E: 7.4 ± 4.9/HY II-IV	NR	C: AD E: AD+CBT	(1) MMSE (2) PDSS (3) HAMD (4) HAMA
Rodgers et al.	2019	China	T: 63.70 ± 8.76	NR	C: 15 E: 12	NR	NR	C: Waiting for control E: M-CBT	(1) PDQ-39
Kalyani et al.	2019	Australia	C: 66.50 ± 7.70 E: 65.24 ± 11.88	C: 10/6 E: 3/14	C: 16 E: 17	C: 5.94 ± 3.61 E: 3.76 ± 2.88/HY I-III	NR/ C: 715.56 ± 418.38 E: 533.00 ± 315.548	C: TAU E: Dance	(1) PDQ-39
Solla P et al.	2019	Italy	C: 67.1 ± 6.3 E: 67.8 ± 5.9	C: 7/3 E: 6/4	C: 10 E: 10	C: 5 ± 2.9 E: 4.4 ± 4.5/HY < III	NR	C: TAU E: BS dance	(1) BDI (2) MoCA
Rios Romenets et al.	2015	Canada	C: 64.3 ± 8.1 E: 63.2 ± 9.9	C: 7/8 E: 12/6	C: 15 E: 18	C: 7.7 ± 4.6 E: 5.5 ± 4.4/HYI-III	Levodopa/ C: 485 ± 347.5 E: 450 ± 349.7	C: Waiting for control E: tango	(1) BDI (2) PDQ-39
Ferreira et al.	2018	Brazil	C: 67.6 ± 8.9 E: 64.1 ± 7.0	NR	C: 17 E: 18	C: 4.5 ± 4.0 E: 6.4 ± 2.7/HYI-III	Levodopa + Carbidopa/C: 5 E: 5	C: SPT E: RT + SPT	(1) PDQ-39
Xi et al.	2022	China	C: 67.02 ± 4.52 E: 67.41 ± 4.42	C: 15/18 E: 17/16	C: 33 E: 33	C: 3.36 ± 1.52 E: 4.00 ± 2.06/HY > III	NR	C: SPR E: ATM + SPR	(1) HAMD (2) PDQ-39
Wu et al.	2018	China	C: 64.66 ± 5.47 E: 62.42 ± 5.37	C: 17/7 E: 20/8	C: 28 E: 24	NR/HYI-III	NR	C: TAU E: Tai Chi + TAU	(1) MoCA (2) PDQ-39
Cao et al.	2021	China	C: 62.97 ± 3.27 E: 62.45 ± 2.87	C: 21/10 E: 20/11	C: 31 E: 31	C: 5.71 ± 1.10 E: 6.10 ± 0.87/HY < III	NR	C: TAU E: Wuqinxi + TAU	(1) PDQ-39
Moon et al.	2020	United States	C: 65.9 ± 5.4 E: 66.4 ± 8.1	C: 3/6 E: 4/4	C: 9 E: 8	C: 5.33 ± 3.3 E: 4.25 ± 2.1/HYI-III	NR/ C: 712.6 ± 332.4 E: 682.6 ± 301.1	C: Sham Qigong E: Qigong	(1) PDSS (2) PDQ-39 (3) MMSE
Zhu et al.	2020	China	C: 67.77 ± 1.72 E: 68.53 ± 1.90	C: 13/9 E: 12/7	C: 22 E: 19	C: 4.00 ± 0.39 E: 4.68 ± 0.43/HYI-III	NR	C: RT E: RT + Tai Chi	(1) HAMD (2) HAMA (3) PDSS (4) MoCA (5) PDQ-39
Wang et al.	2022	China	C1: 66.20 ± 4.08 C2: 67.95 ± 4.86 E: 68.83 ± 4.35	C1: 11/14 C2: 10/12 E: 7/16	C1: 25 C2: 22 E: 23	C1: NR C2: 6.09 ± 3.85 E: 6.63 ± 4.01	Levodopa/ C: 347.73 ± 145.09 E: 315 ± 134.04	C1:no treatment C2: SE E: Wuqinxi	(1) PDSS (2) PDQ-39 (3) MoCA
Cheng et al.	2022	Taiwan-China	C: 73.9 ± 6.9 E1: 71.6 ± 5.1 E2: 71.4 ± 8.5	C: 11/5 E1: 6/5 E2: 7/6	C: 16 E1: 11 E: 13	NR/HY I-IV	Levodopa/ C: 681 ± 324.6 E: 573.6 ± 272.7	C: Sham rTMS E1: 5Hz iTBS rTMS E2: VR-CBT + 5 Hz iTBS rTMS (Left DLPFC)	(1) MoCA (2) BDI
Chen et al.	2022	China	C1: 63.7 ± 8.88 C2: 64.34 ± 9.68 C3: 64.35 ± 8.93 E: 64.17 ± 8.37	C1: 11/18 C2: 12/17 C3: 12/19 E: 12/17	C1: 29 C2: 29 C3: 31 E: 29	C1: 3.79 ± 3.07 C2: 3.59 ± 3.70 C3: 3.71 ± 2.47 E: 4.14 ± 2.47/HY II-IV	Levodopa Benserazide/ C_1–3_: 250 to 1,000 E: 250 to 1,000	C1: TAU C2: TAU + escitalopram C3: TAU + pramipexole E: TAU+5 Hz rTMS (Left DLPFC)	(1) HAMD (2) PDQ-39
Su et al.	2012	China	C: 59.1 ± 4.7 E: 57.3 ± 5.9	C: 13/13 E: 15/14	C: 26 E: 29	C: 6.9 ± 1.7 E: 6.5 ± 1.4/NR	NR	C: Sham rTMS E: 0.5 Hz rTMS (BF + PL)	(1) HAMD (2) PDSS (3) MMSE
Wu et al.	2019	China	C1: 60.5 ± 5.8 E1: 59.6 ± 6.1 E2: 60.2 ± 6.3	C: 28/22 E1: 30/20 E2: 31/19	C: 50 E1: 50 E2: 50	C: 5.5 ± 1.4 E1: 5.8 ± 1.6 E2: 6.0 ± 1.7/HYI-III	NR	C: TAU E: TAU+1/5 Hz rTMS (Left DLPFC)	(1) MMSE (2) HAMD (3) HAMA (4) PDSS
He et al.	2021	Hong Kong	C: 74.8 ± 6.9 E: 70.0 ± 6.3	C: 10/5 E: 13/7	C: 15 E: 20	C: 2.5 ± 1.1 E: 2.7 ± 1.5 /HY-IV	NR	C: Sham iTBS E: iTBS (Left DLPFC)	(1) MoCA (2) BDI
Cardoso et al.	2008	Brazil	C: 63 ± 7.1 E: 67 ± 8.3	NR	C: 10 E: 11	C: 11 ± 6.4 E: 11 ± 7.65/HYI-IV	Levodopa/ C: 1100 E: 975	C: Sham rTMS + fluoxetine E: 5 Hz rTMS + placebo (Left DLPFC)	(1) HAMD (2) BDI (3) MMSE
Li et al.	2020	China	C: 64.46 ± 8.40 E: 61.67 ± 6.92	C: 8/16 E: 8/16	C: 24 E: 24	C: 6.46 ± 5.17 E: 5.48 ± 3.69/HYI-IV	NR/ C: 556.60 ± 423.04 E: 435.29 ± 251.12	C: sham rTMS E: 20 Hz rTMS (M1)	(1) HAMD (2) HAMA (3) PDSS (4) PQD-39

E, experimental group; C, control group; T, total; M, male; F, female; y, years; NR, not reported; HY, Hoehn and Yahr scale; LEDD, Levodopa equivalent daily dose; AT, acupuncture; AD, antiparkinsonian drugs; TAU, treatment as usual; EAT, electroacupuncture; CM, clinical monitoring; CBT, cognitive behavioral therapy; SH, speech therapy; U-CBT, unstructured cognitive behavioral therapy; S-CBT, structured cognitive behavioral therapy; T-CBT, telephone-based cognitive behavioral therapy; SE, stretching exercise; TS, telephone session; SPR, standard physical rehabilitation; rTMS, repetitive transcranial magnetic stimulation; SPT, standard pharmacological treatment; RT, resistance training; ATM, abdominal torsion movement; iTBS, intermittent theta burst stimulation; DLPFC, dorsolateral prefrontal cortex; BF, bilateral frontal; PL, parietal lobes; PDQ-39, Parkinson’s Disease Questionnaire-39; PDSS, Parkinson’s Disease Sleep Scale; BSS, Bristol Stool Scale; CCS, Cleveland Clinic Constipation Score; BDI, Beck Depression Inventory; MoCA, Montreal Cognitive Assessment; HAMD, Hamilton Depression Rating Scale; HAMA, Hamilton Anxiety Rating Scale; MMSE, Minimum Mental State Examination.

### Risk of bias

**Figure 2:**
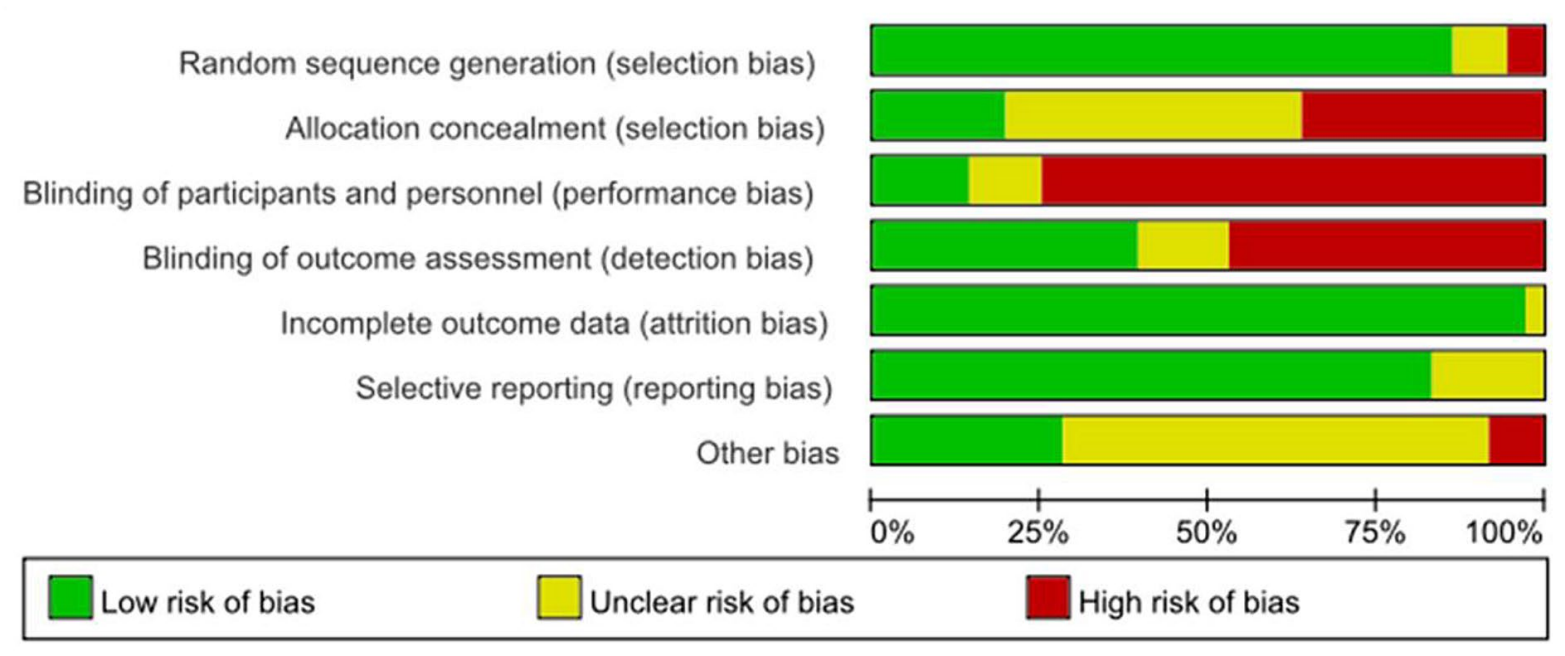
The risk of bias in the included studies.

**Figure 3:**
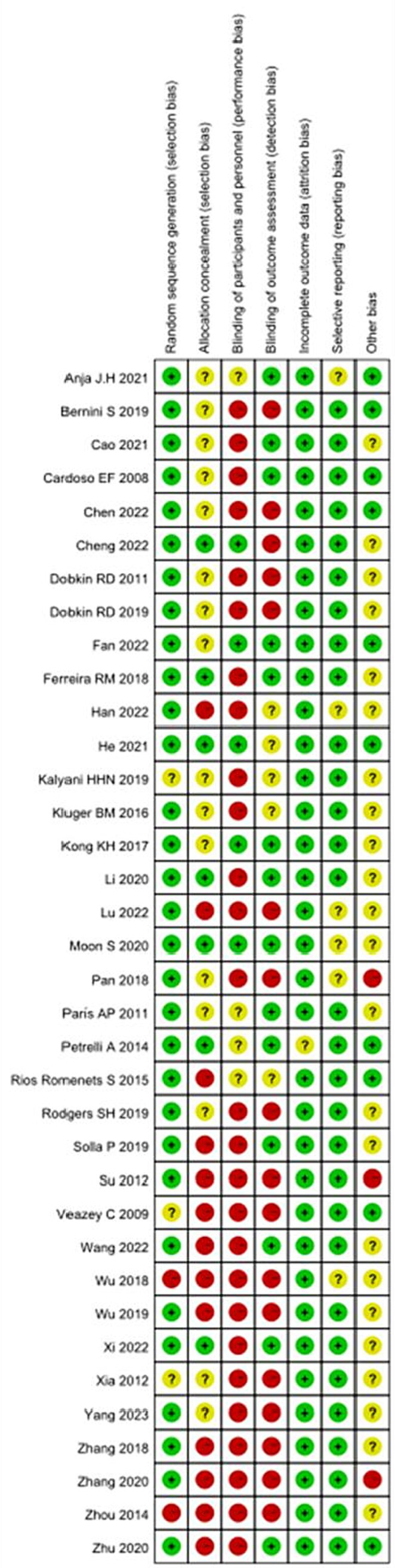
Risk of bias summary: each risk of bias item for each included study. CBT, cognitive behavioral therapy; rTMS, repetitive transcranial magnetic stimulation.

In this review, the Cochrane risk assessment tool (Higgins and Green, 2011) was used to analyze the risk level of the included studies. Among the included studies, 33 studies explained or described the randomization method in detail, and 3 studies only showed the word “randomized” or did not describe the randomization method. Only seven studies described allocation concealment methods in detail. “Because of the specificity of nonpharmacological interventions, blinding of patients or intervenors is difficult, and 27 studies were described as high risk, with unknown risk due to only the use of blinding or uncertainty about the reliability of such interventions in blinding patients.” The included studies either did not drop out patients, or all included detailed reasons for dropout, and the other 23 studies were mainly judged as uncertain risk of bias in terms of other biases because there was not enough information to judge whether there was a significant risk of bias in these studies, see Figures 2, 3.

### Sleep symptoms in PD

**Figure 4:**
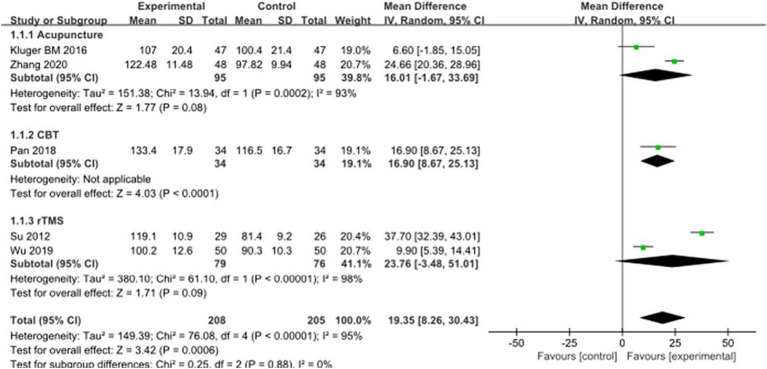
Comparison of non-pharmacological therapies in terms of PDSS. CBT, cognitive behavioral therapy; rTMS, repetitive transcranial magnetic stimulation.

Five studies selected PDSS score to evaluate the improvement of sleep symptoms in PD patients after intervention, involving a total of 413 patients. Compared with the control group, the PDSS score of the experimental group was significantly improved [MD = −19.35, 95% CI (−30.4 to −8.28), *p* < 0.0006], and there was significant heterogeneity (*χ*^2^ = 75.02, *p* < 0.00001, *I*^2^ = 95%). There was no significant difference between the subgroups (*χ*^2^ = 0.25, *p* = 0.88, *I*^2^ = 0%). Subgroup analysis showed significant differences between the CBT subgroup [MD = −16.90, 95% CI (−25.13 to −8.67), *p* < 0.0001] and the control group; there was no significant difference between the acupuncture subgroup [MD = −16.01, 95% CI (−33.69 to 1.67), *p* = 0.08] and the rTMS subgroup [MD = −23.77, 95% CI (−51.01 to 3.48), *p* < 0.09] compared to the control group. The 5 included studies were excluded in turn, there was no significant change in effect size and heterogeneity, suggesting that the combined results were relatively robust. See Figure 4 for details.

### Depressive symptoms in PD

**Figure 5:**
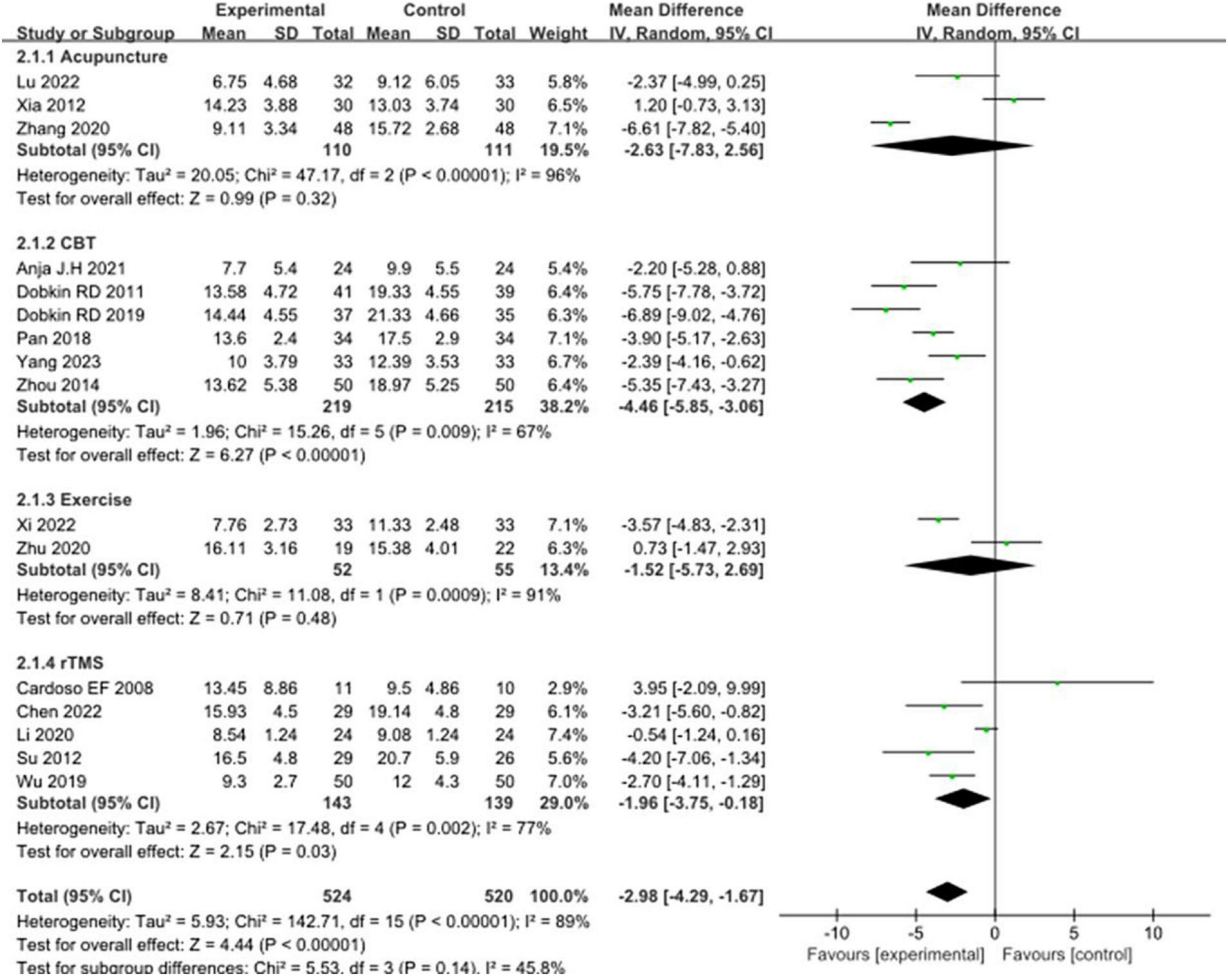
Comparison of non-pharmacological therapies in terms of HAMD. CBT, cognitive behavioral therapy; rTMS, repetitive transcranial magnetic stimulation.

**Figure 6:**
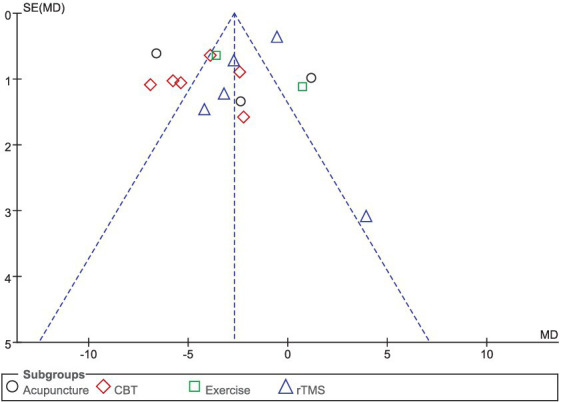
HAMD funnel plot.

Sixteen studies selected HAMD scores to evaluate the improvement of depressive symptoms in PD patients after intervention. A total of 1,044 patients were involved. Compared with the control group, the HAMD score of the experimental group was significantly lower [MD = −2.98, 95% CI (−4.29 to −1.67), *p* < 0.00001], and there was significant heterogeneity (*χ*^2^ = 142.71, *p* < 0.00001, *I*^2^ = 89%). There was no significant difference between the subgroups (*χ*^2^ = 5.53, *p* = 0.14, *I*^2^ = 45.8%). In the subgroup analysis, there was no significant difference between the acupuncture subgroup [MD = −2.63, 95% CI (−7.83 to 2.56), *p* = 0.32] and the control group. CBT [MD = 4.46, 95% CI (5.85 to 3.06), *p* < 0.00001], Exercise [MD = 1.52, 95% CI (5.73 to 2.69), *p* = 0.048], rTMS [MD = 1.96, 95% CI (3.75 to −0.18), *p* = 0.03], there were significant differences compared with the control group. The 16 included studies were excluded sequentially, and the effect size and heterogeneity did not change significantly. Excluding (Li et al., 2020), the effect size and heterogeneity of the rTMS subgroup changed significantly (*I*^2^ change >20%) (*χ*^2^ = 17.48, *p* = 0.12, *I*^2^ = 49%). The effect size and heterogeneity did not change significantly when each subgroup was sequentially excluded according to the subgroup classification unit. Combined with funnel plot analysis, the 16 included studies had minor publication bias in terms of HAMD scores. See Figures 5, 6 for details.

**Figure 7:**
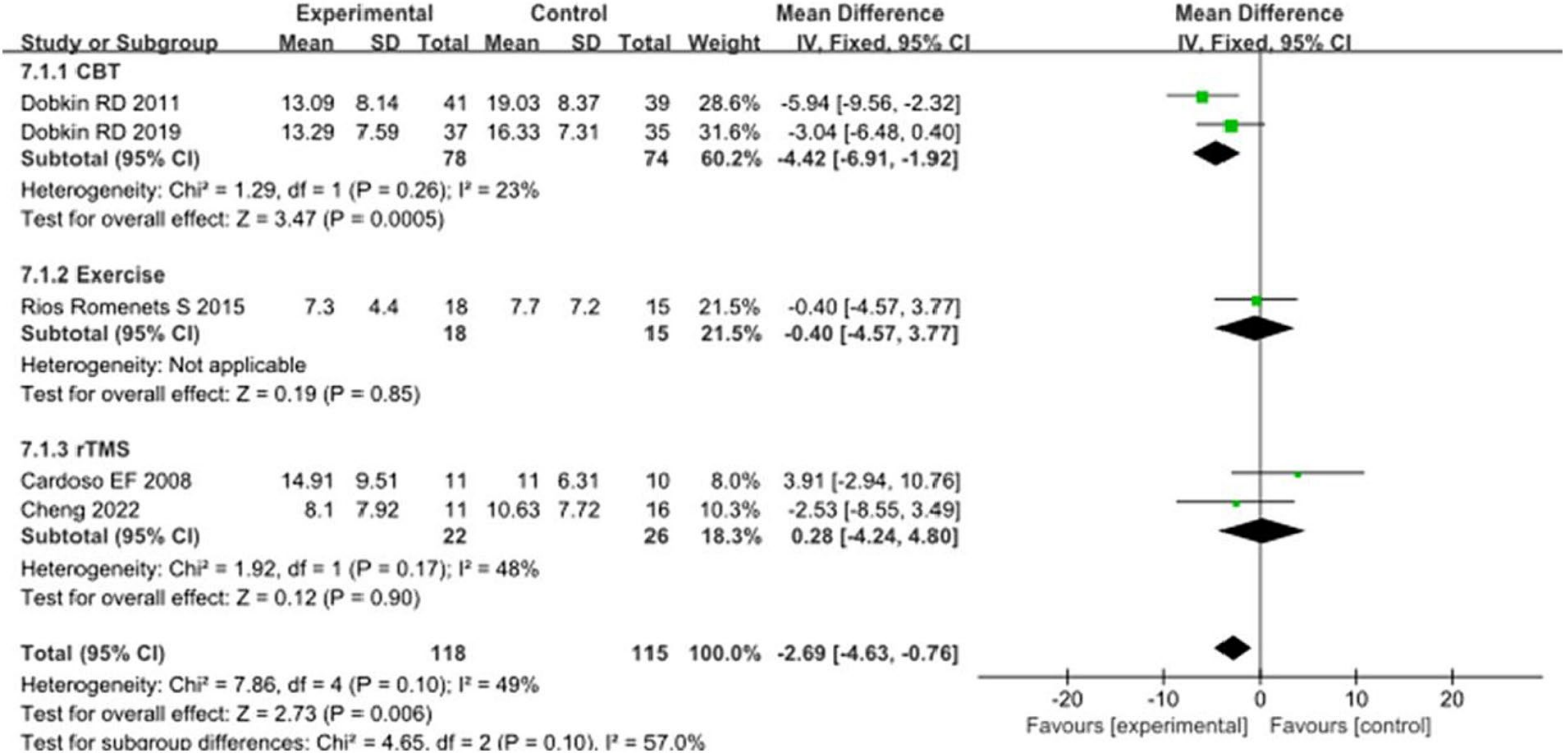
Comparison of non-pharmacological therapies in terms of BDI. CBT, cognitive behavioral therapy; rTMS, repetitive transcranial magnetic stimulation.

In addition, 5 studies selected BDI scores to evaluate the improvement of depressive symptoms in PD patients after intervention. A total of 233 patients were involved. Compared with control group, experimental group BDI score significantly reduced [MD = 2.69, 95% CI (4.24 to 4.80), *p* = 0.006], no significant heterogeneity (*χ*^2^ = 7.86, *p* = 0.1, *I*^2^ = 49%). There was also no significant heterogeneity between subgroups (*χ*^2^ = 4.65, *p* = 0.1, *I*^2^ = 57%). In the subgroup analysis, there was a significant difference between the CBT subgroup [MD = −4.42, 95% CI = (−6.91 to −1.92), *p* = 0.0005] and the control group. Exercise [MD = 0.40, 95% CI (4.57 to 3.77), *p* = 0.85], rTMS [MD = 0.28, 95% CI (4.24 to 4.80), *p* = 0.90], there was no significant difference compared with the control group. The 5 included studies were excluded in turn, there was no significant change in effect size and heterogeneity. After replacing the random effect model, the effect size of the experimental group [MD = −2.26, 95% CI (−5.10 to 0.58), *p* = 0.12] was not significantly different from that of the control group. The combined results of the two combined effect models were inconsistent, suggesting the lack of robustness of the combined results. See Figure 7 for details.

### Anxiety symptoms in PD

**Figure 8:**
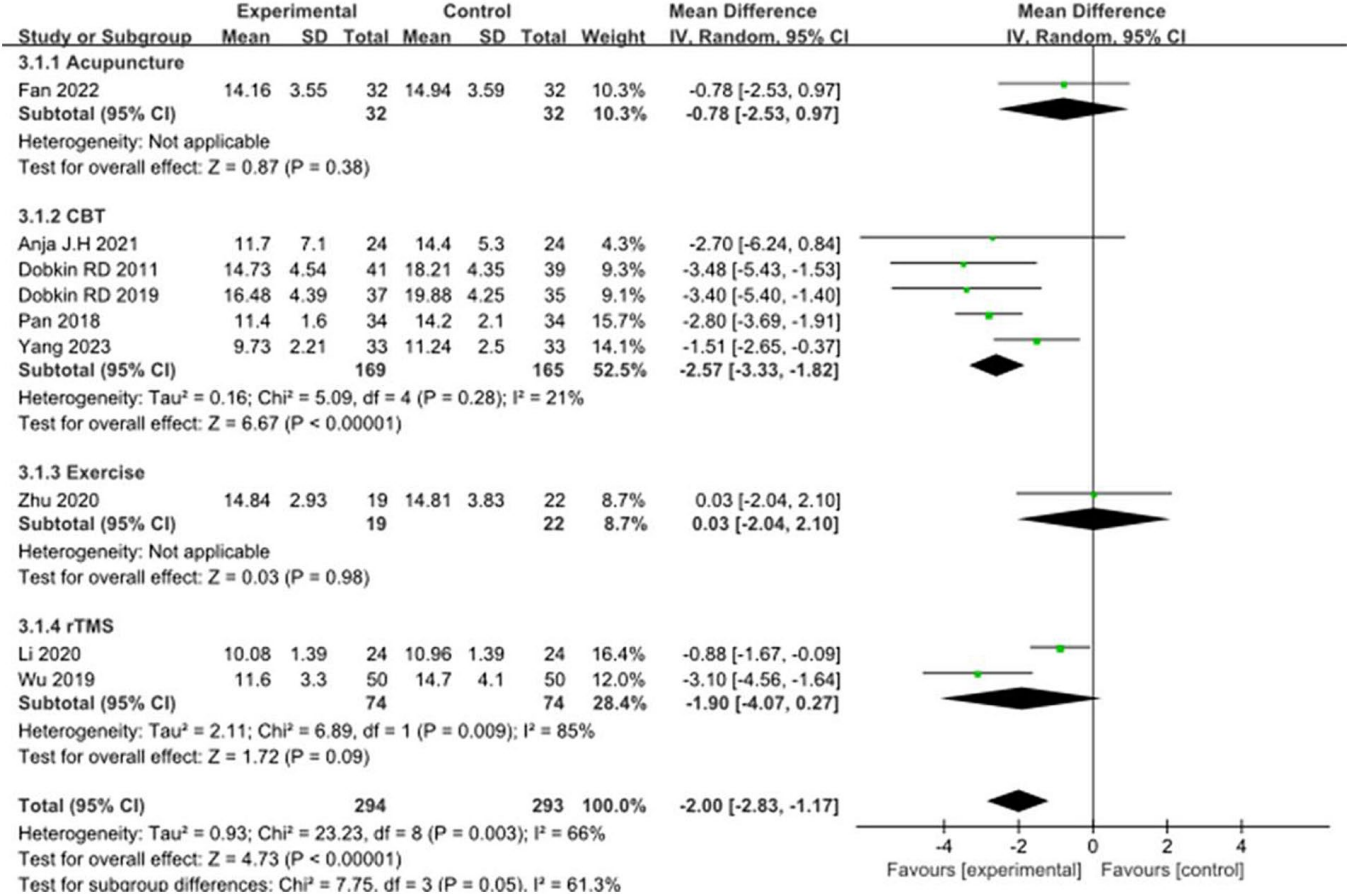
Comparison of non-pharmacological therapies in terms of HAMA. CBT, cognitive behavioral therapy; rTMS, repetitive transcranial magnetic stimulation.

Nine studies selected the HAMA score to evaluate the improvement in anxiety symptoms in PD patients after the intervention. A total of 587 patients were involved. Compared with the control group, the HAMA score was significantly lower [MD = −2.00, 95%CI (−2.83 to −1.17), *p* < 0.00001] with some heterogeneity (*χ*^2^ = 23.23, *p* = 0.003, *I*^2^ = 66%). There was also some heterogeneity between the subgroups (*χ*^2^ = 7.75, *p* = 0.05, *I*^2^ = 61.3%). Subgroup analysis revealed that Acupuncture subgroup [MD = −0.78, 95% CI (−2.53 to 0.97), *p* = 0.38], Exercise Subgroup [MD = -0.03, 95% CI (−2.04 to 2.10), *p* = 0.98], rTMS subgroup [MD = 1.90, 95% CI (−4.07 to 0.27), *p* = 0.09], there was no significant difference compared with the control group; The CBT subgroup [MD = −2.57, 95% CI (−3.33 to −1.82), *p* = 0.0001], significant differences were observed when compared with the control group. The 9 included studies were excluded sequentially, exclusion (Li et al., 2020), combined effect size [MD = −2.22, 95% CI (−3.05 to −1.39), *p* < 0.00001] and heterogeneity (*χ*^2^ = 14.60, *p* = 0.04, *I*^2^ = 52%) changed significantly. This suggests that the combined results lack robustness. See Figure 8 for details.

### Cognitive symptoms in PD

**Figure 9:**
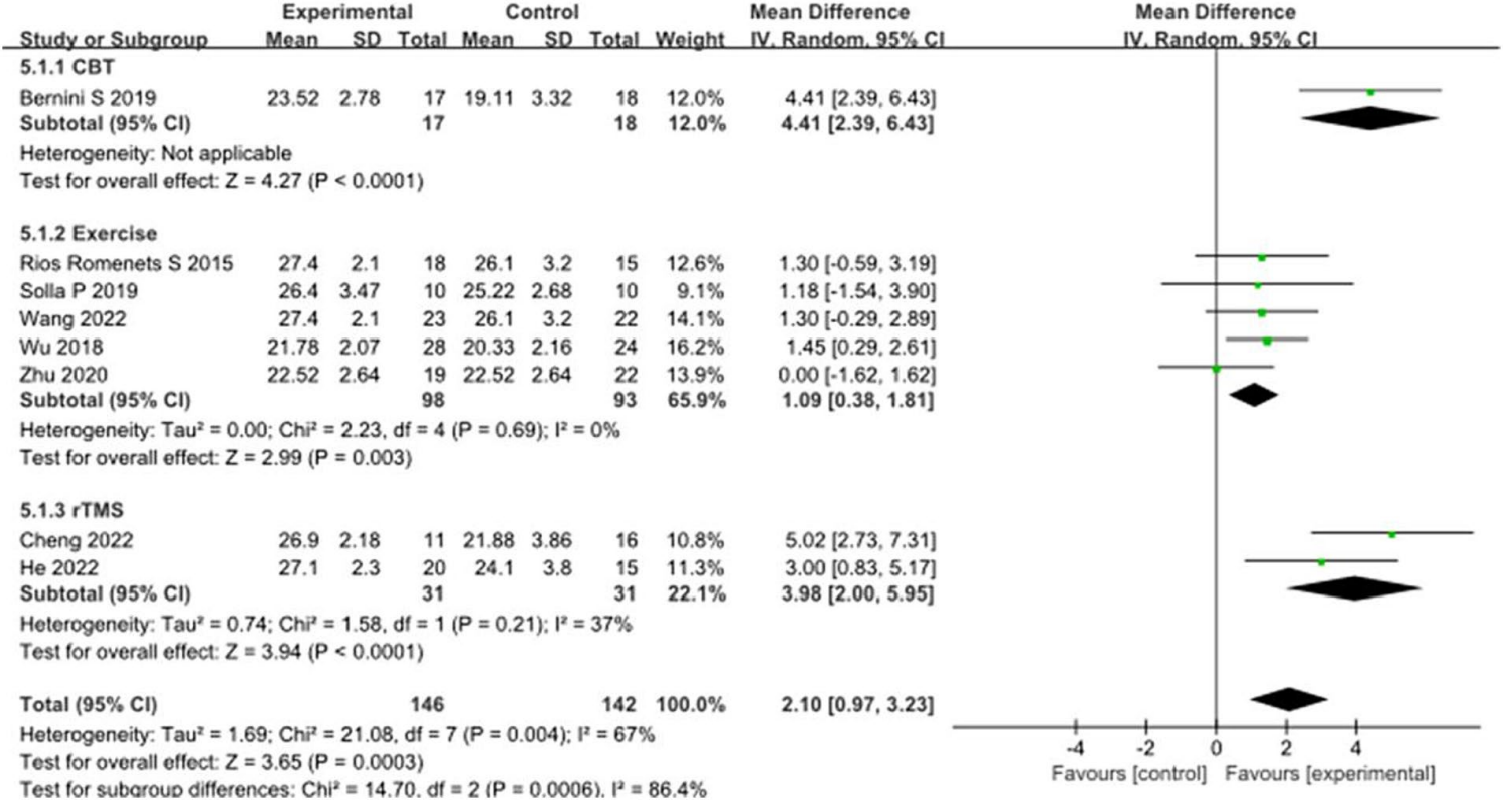
Comparison of non-pharmacological therapies in terms of CoMA. CBT, cognitive behavioral therapy; rTMS, repetitive transcranial magnetic stimulation.

CoMA scores, as one of the main cognitive assessment tools for PD patients, eight studies selected CoMA scores to evaluate the improvement of cognitive function in PD patients after intervention. A total of 288 patients were involved. Compared with the control group, the CoMA score of the experimental group was significantly higher [MD = 2.10, 95% CI (−0.97 to 3.23), *p* = 0.0003], and there was significant heterogeneity (*χ*^2^ = 21.08, *p* = 0.004, *I*^2^ = 67%). There were significant differences between subgroups (*χ*^2^ = 14.70, *p* = 0.0006, *I*^2^ = 86.4%). Subgroup analysis showed that significant differences existed between the CBT subgroups [MD = 4.41, 95%CI (2.39 to 6.43), *p* < 0.0001], Exercise subgroups [MD = 1.09, 95% CI (0.38 to 1.81), *p* = 0.003], rTMS subgroups [MD = 3.98, 95% CI (2.00 to 5.95), *p* < 0.0001] compared to the control group. The 8 studies that were included were excluded sequentially, and the effect size and heterogeneity did not change significantly. This suggests that the pooled results are robust. See Figure 9 for details.

**Figure 10:**
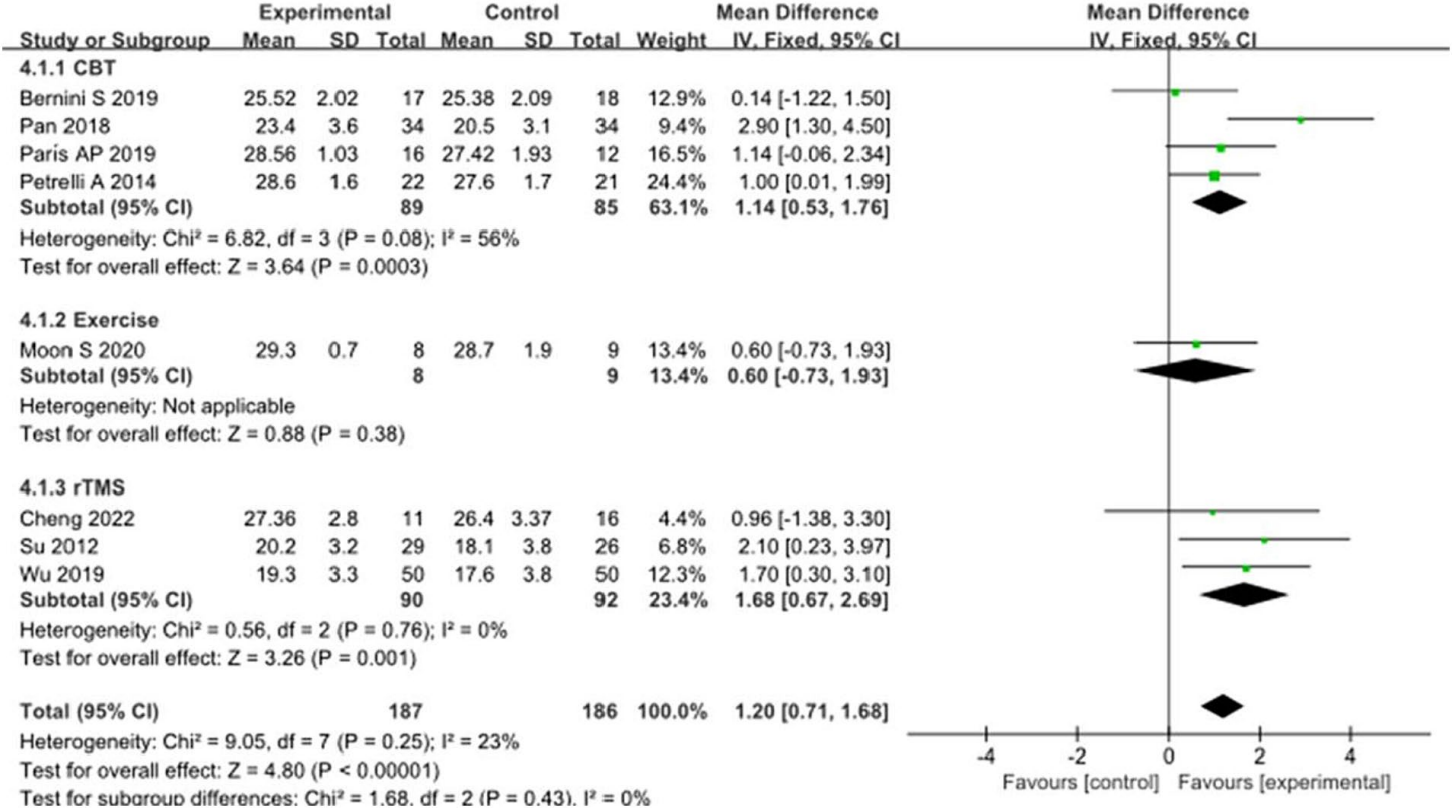
Comparison of non-pharmacological therapies in terms of MMSE. CBT, cognitive behavioral therapy; rTMS, repetitive transcranial magnetic stimulation.

In addition, eight studies selected MMSE scores to evaluate the recovery of cognitive function in PD patients after intervention. A total of 373 patients were involved. Compared with the control group, the MMSE score of the experimental group was significantly improved [MD = 1.20, 95% CI (0.71 to 1.68), *p* < 0.00001], and there was no significant heterogeneity (*χ*^2^ = 9.05, *p* = 0.25, *I*^2^ = 23%). There was no heterogeneity between subgroups (*χ*^2^ = 1.68, *p* = 0.43, *I*^2^ = 0%). Subgroup analysis showed significant differences between the CBT subgroup [MD = 1.14, 95% CI (0.53 to 1.76), *p* = 0.0003] and the rTMS subgroup [MD = 1.68, 95% CI (0.67 to 2.69), *p* = 0.001] compared to the control group; There was no significant difference between the exercise subgroup [MD = 0.60, 95% CI (−0.73 to 1.93), *p* = 0.38] and the control group. The 8 studies that were included were excluded sequentially, and the effect size and heterogeneity did not change significantly. After changing the random effect type, the effect size of the experimental group [MD = 1.22, 95% CI (0.66 to 1.79), *p* < 0.0001] still showed significant differences compared to the control group, with no significant heterogeneity (*χ*^2^ = 9.05, *p* = 0.25, *I*^2^ = 23%), indicating that the merged results are relatively robust. See Figure 10 for details.

### Quality of life in PD

**Figure 11:**
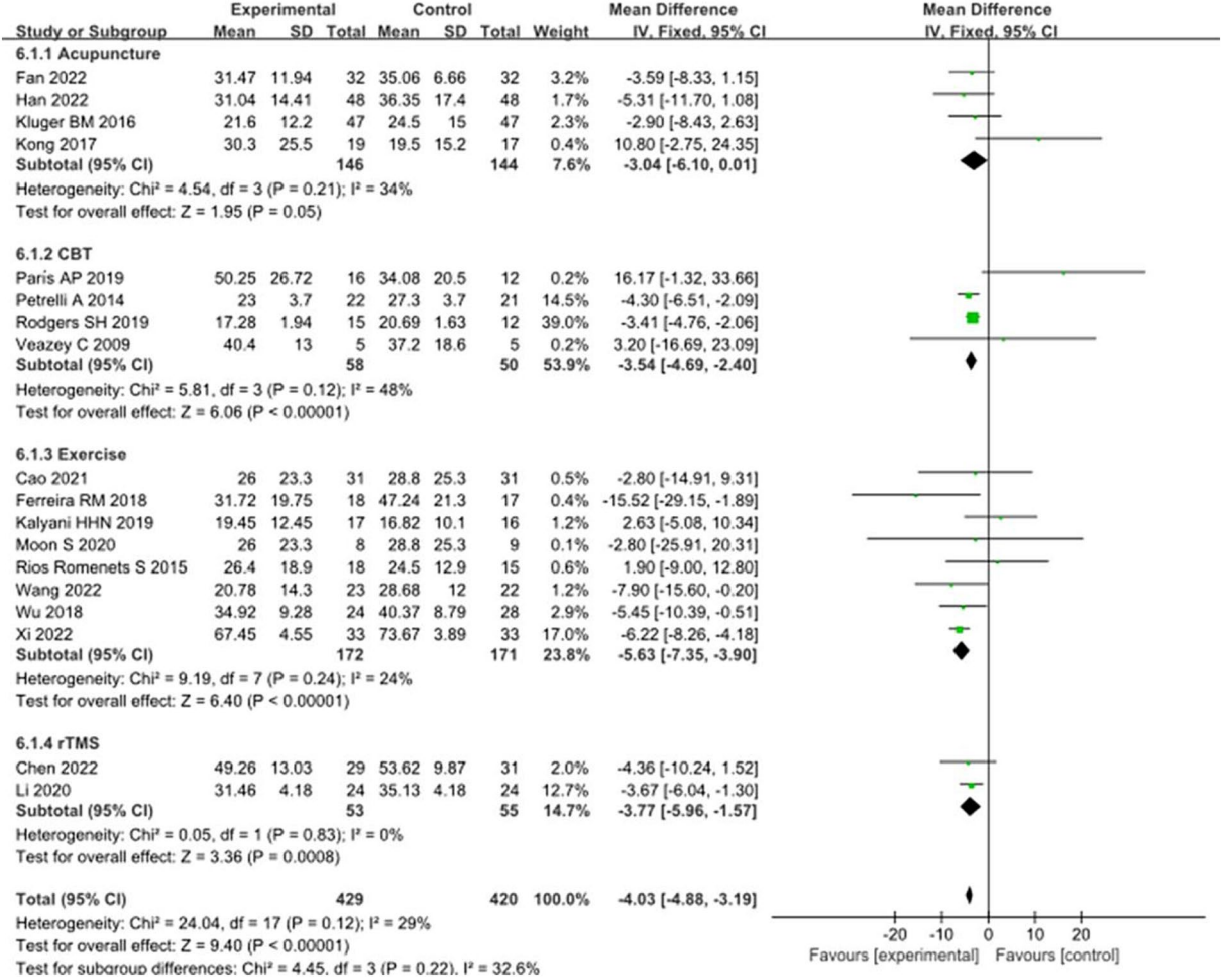
Comparison of non-pharmacological therapies in terms of PDQ-39.

**Figure 12:**
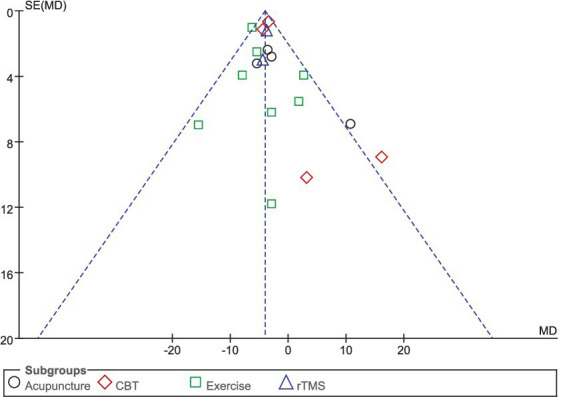
PDQ-39 funnel plot.

Eighteen studies selected the PDQ-39 score to assess the improvement of quality of life in PD patients after intervention. A total of 849 patients were involved. Compared with control group, experimental group PDQ - 39 score decreased significantly [MD = 4.03, 95% CI (5.96 to 1.57), *p* < 0.00001], no significant heterogeneity (*χ*^2^ = 24.04, *p* = 0.12, *I*^2^ = 29%). There was also no significant heterogeneity among the subgroups (*χ*^2^ = 4.45, *p* = 0.22, *I*^2^ = 32.6%). Subgroup analysis showed that compared with the control group, the acupuncture subgroup [MD = −3.04, 95% CI (−6.10 to 0.01), *p* = 0.05], CBT subgroups [MD = 3.54, 95% CI (4.69 to 2.40), *p* < 0.00001], and Exercise subgroups [MD = 5.63, 95% CI (7.35 to 3.90), *p* < 0.00001], rTMS subgroups [MD = 3.77, 95% CI (5.96 to 1.57), *p* = 0.0008], there were significant differences. The 8 studies that were included were excluded sequentially, and the effect size and heterogeneity did not change significantly. After replacing the random effect type, the effect size of the experimental group [MD = −3.96, 95% CI (−5.26 to −2.66), *p* < 0.00001] was significantly different from that of the control group, and the heterogeneity (*χ*^2^ = 24.04, *p* = 0.12, *I*^2^ = 29%) suggested that the combined results were more robust. Combined with funnel plot analysis, the 18 included studies had minor publication bias in terms of PDQ-39 scores. See Figures 11, 12 for details.

### Qualitative analysis

Zhang et al. (2018) used acupuncture to treat constipation in PD patients. The control group was treated with electroacupuncture, and the experimental group was treated with conventional acupuncture at Zhigou (TE6) and Zhaohai (KI6). Bristol Stool Scale (Bristol Stool Scale) and Cleveland Clinic Constipation Score (CCS) were used to evaluate the symptoms of constipation after the intervention. After 24 treatments, the two scores in the experimental group were significantly different from the baseline comparison and significantly different from the control group, suggesting that acupuncture is effective for constipation. However, in terms of treating constipation symptoms in PD patients, only one study met the inclusion requirements, so the study was evaluated qualitatively.

## Discussion

Studies have shown that non-drug interventions such as complementary or alternative therapy are increasingly used to treat motor symptoms and non-motor symptoms in patients with PD (Ghaffari and Kluger, 2014). Previous reviews primarily focused on specific non-drug means or mainly focused on motor symptoms. This meta-analysis and systematic review comprehensively evaluated four common non-drug treatments, and there were significant improvements in 8 outcomes. Due to the particularity of acupuncture and exercise intervention, it is challenging to implement in the field of the blind method. However, in other areas, the four interventions reflect a higher research quality, which helps reduce the methodological heterogeneity of the research. However, this study focused on the effect of non-drug therapy on PD patients, and there is a lack of large-scale and high-quality research support in this direction. Moreover, most of the literature sources of non-motor therapy such as acupuncture and exercise are from China, which may have regional bias.

### Acupuncture

Of the 8 acupuncture studies included, 7 used conventional acupuncture, and our analysis focused on the effectiveness of this type of acupuncture within the subgroups. In the acupuncture subgroup, we found that the HAMD and HAMA of the experimental group had a certain improvement trend but did not reach statistical significance. A possible reasonable explanation is the placebo effect of acupuncture in a short time. In the (Fan et al., 2022) study, there was no statistical difference in HAMA score between the treatment group and the sham acupuncture group, but its effectiveness was determined by the minimum difference of clinical significance. There was a significant statistical difference between the two groups during the follow-up 2 months later. Due to the high recognition of acupuncture efficacy in China, sham acupuncture participants included in the study may think that they have received adequate treatment, so there may be a significant placebo effect in the course of treatment. With the decrease of the placebo effect, the long-term benefits of acupuncture can maintain the efficacy of the acupuncture group. In addition, this study validates the effectiveness of acupuncture in reducing ACTH (adreno-cortico-tropic-hormone, ACTH) levels, indirectly indicating the efficacy of acupuncture on anxiety symptoms (Zhang et al., 2018; Xi and Bi, 2022) there is a strict restriction on the HAMD score of patients included in PD (24 > HAMD > 7), which is beneficial for reducing clinical heterogeneity. Still, the slightly different intervention methods of the three studies included, and the low quality of the research methodology may be the reasons for the poor statistical results of HAMD after the merger. In the acupuncture subgroup, we found that acupuncture significantly improved the PDQ-39 score of PD patients, and the subset was homogeneous. Acupuncture belongs to traditional Chinese treatment, an essential part of complementary or alternative therapy. The efficacy of acupuncture in various primary or secondary nervous system diseases may be extended to the PD population. A large number of studies have proved that acupuncture-related therapy can significantly improve the motor symptoms of PD. Still, non-motor symptoms are often ignored as a secondary result, resulting in the lack of related large samples and high-quality RCT (Deuel and Seeberger, 2020). In this meta-analysis, acupuncture can be used as a potential non-pharmacological treatment, but the reliability of the combined results needs to be further verified by a large number of high-quality studies.

### CBT

Cognitive behavioral therapy (CBT) is a kind of psychotherapy that can improve mental health by changing negative thinking patterns and behavior habits. CBT usually consists of two parts: cognitive therapy and behavioral therapy. A large number of studies have used CBT to treat psychological problems, such as anxiety, depression, post-traumatic stress disorder, obsessive-compulsive disorder, panic disorder, and food and drug abuse. Of the 11 studies included in this review, 5 trials used CBT tailored to the specific needs of PD patients, 5 tests used structured CBT therapy (including structured exercise, behavioral activation, thought monitoring and reorganization, relaxation training, anxiety control and sleep hygiene), and another study did not describe the specific content of CBT. Among the 11 studies in our review, CBT intervention significantly improved insomnia, anxiety, depression, cognition, and quality of life in patients with PD compared with the control group. Subgroup analysis shows that CBT has homogeneity in BDI score, HAMA score, and PDQ-39 score due to the relative consistency of CBT treatment procedures in each CBT subgroup, which will effectively ensure a low clinical heterogeneity among the research evidence. In our screened study, there are few studies on the application of PDSS and CoMA scores by CBT, which may be due to the selection of other evaluation criteria to evaluate sleep and cognitive problems. The sensitivity analysis of the CBT subgroup in the HAMD score of the depression scale was carried out, and the heterogeneity did not change significantly after removing the literature. There was no statistical difference between the CBT intervention and control groups, indicating that the combined result was robust. The sensitivity analysis of the CBT subgroup in the MMSE score of the cognitive scale was carried out after excluding the study (Pan, 2018). The CBT subgroup showed homogeneity [*χ*^2^ = 1.35, MD = 0.84, 95% CI = (0.17 to 1.50), *I*^2^ = 0]. We believe that in the study (Pan, 2018), the experimental group CBT therapy combined with Western medicine pramipexole may have achieved more excellent therapeutic benefits than other studies. In this review, we found that CBT is an effective treatment to reduce PD anxiety and depression and improve the quality of life. PD patients should be encouraged to choose CBT intervention for non-motor symptoms. In addition, there are few studies on PD patients with insomnia or cognitive impairment by CBT, and more and more in-depth studies are needed to evaluate its reliability.

### Exercise

Exercise therapy includes various forms of exercise such as walking, running, yoga, dance and traditional Chinese exercise methods such as Tai Chi, Qigong, Wuqinxi and so on. In previous meta analysis, exercise is considered to be a potentially effective method for the treatment of motor symptoms in PD. In the meta-analysis of Ernst et al. (2023), the combined Parkinson’s Disease rating scale (United Parkinson’s Disease Rating Scale, UPDRS) was used to explain the effect of exercise training on PD participants, and proved that this training was effective for PD patients (Tang et al., 2019). Meta-analysis evaluated the effectiveness of exercise therapy for PD and suggested that tango could significantly enhance functional activity in patients with PD. In our meta-analysis, there were significant differences only in CoMA and PDQ-39 scores between the exercise and control groups. The efficacy of exercise in anxiety scale HAMA score, Depression scale HAMD, BDI score, Cognition scale MMSE score was not accurate. There are few studies on exercise therapy using the above scale, so it is difficult to make a positive evaluation of the efficacy of exercise therapy on PD non-motor symptoms, including anxiety, depression, cognition, and so on. In the subgroup of exercise therapy, the two scores of CoMA and PDQ-39 showed homogeneity. We analyzed five included studies that selected CoMA scores to evaluate cognitive function, including yoga, dance, Wuqinxi, and Tai Chi, which are essentially multi-task exercises, including not only simple body movements or movements such as walking and turning but also need to understand and learn the exercise patterns and integrate the exercise to achieve coordination and consistency. This requires the participation of cognitive function, which may help improve cognitive function in patients with PD. The improvement of the PDQ-39 scale may be due to the indirect benefit of exercise on improving motor symptoms. The effect of traditional Chinese exercise therapy such as Tai Chi, Qigong and Baduanjin on the non-motor symptoms of PD is not definite at present, and large-scale and high-quality clinical trials are still needed to support it. However, we cannot deny the positive effect of traditional Chinese exercise therapy on PD at this stage.

### rTMS

Previous studies have shown that the mechanism of rTMS in the treatment of non-motor symptoms of PD may be achieved by regulating the levels of neurotransmitters in the brain, such as dopamine and glutamate (Prange et al., 2022). Also in degenerative lesions, rTMS can also drive non-motor symptoms such as memory processes by regulating central nervous system cholinergic activity (Bonnì et al., 2017). In our review, the PDSS, HAMD, CoMA, MMSE, PDQ-39 score of rTMS therapy was significantly better than that of the control group, but the improvement in PDSS, BDI, HAMA score was not significant. There is no difference in the PDSS score between the rTMS subgroup and the control group, and there is significant heterogeneity, we believe that the stimulation prescription (including stimulation frequency, location, time, etc.) selected by the two studies in the subgroup during rTMS intervention is inconsistent, and in the (Wu et al., 2019) study, the basic treatment given to patients includes drug treatment for the corresponding symptoms, which will lead to differences in efficacy and heterogeneity. Interestingly, in the (Cardoso et al., 2008) study, the difference between HAMD, BDI and baseline in the rTMS group and the Fluoxetine group after treatment was reported, but the difference between the two groups was not taken seriously because the authors chose a positive control rather than a placebo. The authors explained that the main purpose of this study was to observe the correlation between depressive symptoms and different regions of the neural network in patients with PD treated with rTMS, rather than to test its efficacy. In addition, another limitation given in the study is that the fake rTMS program is not yet mature at that time. In the HAMA score, rTMS also showed strong heterogeneity. We believe that the heterogeneity is due to the fact that we selected the first treatment node when we selected the (Li et al., 2020) data, while in this study, the first treatment node was only treated for 1 week. This is quite different from the treatment time of other studies included. In addition, studies have shown that the MMSE scale has low sensitivity in assessing cognitive function (Jiang et al., 2020), and there is no direct evidence that TMS can significantly improve the MMSE scale. Although MMSE cannot be ignored, this result still needs to be treated with caution. Due to the small number of literature included, we did not conduct a sensitivity analysis in the subgroup.

## Conclusion

In recent years, more and more clinicians and scientists have realized that the non-motor symptoms of PD are the main factor for the decline of the quality of life of PD patients. Therefore, finding effective and safe treatments for non-motor symptoms is a top priority. To the best of our knowledge, this is the first meta-analysis that synthesizes the efficacy of non-drug interventions on non-motor symptoms in patients with PD. The four non-drug methods used in our review showed extraordinary performance in sleep, depression, anxiety, cognition, constipation, and quality of life. It was mentioned in this review that all non-drug treatments were safe and well tolerated, and no serious adverse events were reported in the included research evidence. We found some differences among the subgroups of different intervention methods. Still, we should carefully explain these results due to the inclusion of less literature in the subgroup and the comparison being more indirect. Therefore, we recommend extensive and rigorous RCT to compare different interventions or further network meta-analysis directly.

## Data availability statement

The raw data supporting the conclusions of this article will be made available by the authors, without undue reservation.

## Author contributions

YuZ: Conceptualization, Data curation, Formal analysis, Funding acquisition, Investigation, Methodology, Project administration, Resources, Software, Supervision, Validation, Visualization, Writing – original draft, Writing – review & editing. SL: Conceptualization, Data curation, Formal analysis, Funding acquisition, Investigation, Methodology, Project administration, Resources, Software, Supervision, Validation, Visualization, Writing – review & editing. YB: Supervision, Conceptualization, Writing – review & editing. KX: Methodology, Writing – review & editing. YaZ: Visualization, Writing – review & editing. YS: Investigation, Writing – original draft. ZL: Methodology, Writing – original draft. SW: Conceptualization, Data curation, Formal analysis, Funding acquisition, Investigation, Methodology, Project administration, Resources, Software, Supervision, Validation, Visualization, Writing – review & editing.
