# Supplementary material for: Non-pharmacological therapies for treating non-motor symptoms in patients with Parkinson’s disease: a systematic review and meta-analysis
Source: Front Aging Neurosci. 2024 Apr 26;16:1363115. doi: 10.3389/fnagi.2024.1363115 (PMC11082280; doi:10.3389/fnagi.2024.1363115)
Supplement: Supplementary file 1 [file Data_Sheet_1.docx]

Supplementary Material

**1.Literature search model**

**Cochrane 935**

Search Name: Cochrane

Last Saved: 12/01/2023 21:18:40

Comment:

ID Search

#1 Parkinson disease

#2 MeSH descriptor: [Parkinson Disease] explode all trees

#3 Non-pharmacological therapies OR non-drug therapies OR acupuncture OR repetitive transcranial magnetic stimulation OR cognitive therapy OR cognitive behavior therapy OR exercise therapy OR Tai Ji OR Qigong OR dance

#4 Randomized Controlled Trial OR randomized OR RCT

#5 #1 AND #2 AND #3 AND #4 935

**Pubmed 886**

Search number Query Sort By Filters Search Details Results Time

7((("Parkinson Disease"[Mesh]) OR ((((Parkinson's Disease) OR (Parkinsonism)) OR (shaking palsy)) OR (Lewy Body Parkinson's Disease))) AND (((((((((((Non-pharmacological therapies) OR (non-drug therapies)) OR (acupuncture)) OR (repetitive transcranial magnetic stimulation)) OR (cognitive therapy)) OR (cognitive behavior therapy)) OR (exercise therapy))) OR ("Tai Ji"[Mesh])) OR ("Qigong"[Mesh])) OR (dance))) AND (randomized controlled trial[Publication Type] OR randomized[Title/Abstract] OR placebo[Title/Abstract]) Filter：Randomized Controlled Trial 866 5:34:31

6((("Parkinson Disease"[Mesh]) OR ((((Parkinson's Disease) OR (Parkinsonism)) OR (shaking palsy)) OR (Lewy Body Parkinson's Disease))) AND (((((((((((Non-pharmacological therapies) OR (non-drug therapies)) OR (acupuncture)) OR (repetitive transcranial magnetic stimulation)) OR (cognitive therapy)) OR (cognitive behavior therapy)) OR (exercise therapy))) OR ("Tai Ji"[Mesh])) OR ("Qigong"[Mesh])) OR (dance))) AND (randomized controlled trial[Publication Type] OR randomized[Title/Abstract] OR placebo[Title/Abstract]) 1,585 23:16:06

5randomized controlled trial[Publication Type] OR randomized[Title/Abstract] OR placebo[Title/Abstract] 1,028,109 23:00:33

4((((((((((Non-pharmacological therapies) OR (non-drug therapies)) OR (acupuncture)) OR (repetitive transcranial magnetic stimulation)) OR (cognitive therapy)) OR (cognitive behavior therapy)) OR (exercise therapy))) OR ("Tai Ji"[Mesh])) OR ("Qigong"[Mesh])) OR (dance) 366,613 22:44:50

3("Parkinson Disease"[Mesh]) OR ((((Parkinson's Disease) OR (Parkinsonism)) OR (shaking palsy)) OR (Lewy Body Parkinson's Disease)) 169,155 22:17:26

2(((Parkinson's Disease) OR (Parkinsonism)) OR (shaking palsy)) OR (Lewy Body Parkinson's Disease) 169,155 22:16:00

1"Parkinson Disease"[Mesh] 82,817 22:09:16

**Embase 136**

Session Results

.......................................................

No. Query Results Results Date

#6. #3 AND #4 AND #5 136 01 Dec 2023

#5. 'randomized controlled trial':ab,ti OR 1,168,090 01 dec 2023

'randomized':ab,ti OR 'placebo':ab,ti OR

'rct':ab,ti

#4. ((((('non pharmacological' AND therapies OR 19,887 01 Dec 2023

'acupuncture'/exp OR acupuncture OR repetitive)

AND transcranial AND magnetic AND

('stimulation'/exp OR stimulation) OR cognitive)

AND ('therapy'/exp OR therapy) OR cognitive) AND

('behavior'/exp OR behavior) AND ('therapy'/exp

OR therapy) OR 'exercise'/exp OR exercise) AND

('therapy'/exp OR therapy) OR tai) AND ji OR

'qigong'/exp OR qigong OR 'dance'/exp OR dance

#3. #1 OR #2 217,708 01 Dec 2023

#2. 'parkinsons disease':ab,ti OR 29,900 01 Dec 2023

'parkinsonism':ab,ti

#1. 'parkinson disease'/exp OR 'parkinson disease' 202,880 01 Dec 2023

**CBM 806**

序号 检索表达式 命中文献数 检索时间

1) "帕金森病"[不加权:扩展] 105862 2023-12-01 21:46:40.0

2) "震颤麻痹"[常用字段:智能] 163846 2023-12-01 21:47:46.0

3) "非药物"[常用字段:智能] OR "针刺"[常用字段:智能] OR "灸法"[常用字段:智能] OR "重复经颅磁"[常用字段:智能] OR "认知疗法"[常用字段:智能] OR "认知行为训练"[常用字段:智能] OR "运动疗法"[常用字段:智能] OR "太极"[常用字段:智能] OR "气功"[常用字段:智能] OR"舞蹈"[常用字段:智能] 333533 2023-12-01 21:52:10.0

4) "随机对照试验"[常用字段:智能] OR "随机"[常用字段:智能] OR "临床观察"[常用字段:智能] OR "疗效观察"[常用字段:智能] 2697142 2023-12-01 21:54:35.0

5) ((("随机对照试验"[常用字段:智能] OR "随机"[常用字段:智能] OR "临床观察"[常用字段:智能] OR "疗效观察"[常用字段:智能]) AND ("非药物"[常用字段:智能] OR "针刺"[常用字段:智能] OR "灸法"[常用字段:智能] OR "重复经颅磁"[常用字段:智能] OR "认知疗法"[常用字段:智能] OR "认知行为训练"[常用字段:智能] OR "运动疗法"[常用字段:智能] OR "太极"[常用字段:智能] OR "气功"[常用字段:智能] OR"舞蹈"[常用字段:智能]) AND ("震颤麻痹"[常用字段:智能]) AND ("帕金森病"[不加权:扩展])) AND (("随机对照试验"[常用字段:智能] OR "随机"[常用字段:智能] OR "临床观察"[常用字段:智能] OR "疗效观察"[常用字段:智能]) AND ("非药物"[常用字段:智能] OR "针刺"[常用字段:智能] OR "灸法"[常用字段:智能] OR "重复经颅磁"[常用字段:智能] OR "认知疗法"[常用字段:智能] OR "认知行为训练"[常用字段:智能] OR "运动疗法"[常用字段:智能] OR "太极"[常用字段:智能] OR "气功"[常用字段:智能] OR"舞蹈"[常用字段:智能]) AND ("震颤麻痹"[常用字段:智能]) AND ("帕金森病"[不加权:扩展]))) 806 2023-12-01 21:55:39.0

**CNKI 481**

（主题：帕金森病(精确)）OR（主题：震颤麻痹(精确)）AND（主题：非药物(精确)）OR（主题：针刺(精确)）OR（主题：灸法(精确)）OR（主题：重复经颅磁(精确)）OR（主题：认知疗法(精确)）OR（主题：认知行为训练(精确)）OR（主题：运动疗法(精确)）OR（主题：太极(精确)）OR（主题：气功(精确)）OR（主题：舞蹈(精确)）AND（摘要：随机对照(精确)）OR（摘要：随机(精确)）OR（摘要：临床观察(精确)）OR（摘要：疗效观察(精确)）

Parkinsons disease parkinsonism Parkinson disease

'Parkinsons disease':ab,ti OR 'parkinsonism':ab,ti

randomized controlled trial OR randomized OR placebo OR RCT

'randomized controlled trial ':ab,ti OR 'randomized':ab,ti OR ' placebo ':ab,ti OR 'RCT':ab,ti

**WANGFANG 404**

主题:(帕金森病 OR 震颤麻痹) and 主题:(非药物治疗 OR 针刺 OR 灸法 OR 重复经颅磁 OR 认知疗法 OR 认知行为训练 OR 运动疗法 OR 太极 OR 气功 OR 舞蹈) and 主题:(随机对照试验 OR 随机 OR 临床观察

**VIP 379**

[(((题名或关键词=帕金森病 OR 题名或关键词=震颤麻痹) AND (((((((((题名或关键词=非药物 OR 题名或关键词=针刺) OR 题名或关键词=灸法) OR 题名或关键词=重复经颅磁) OR 题名或关键词=认知疗法) OR 题名或关键词=认知行为训练) OR 题名或关键词=运动疗法) OR 题名或关键词=太极) OR 题名或关键词=气功) OR 题名或关键词=舞蹈)) AND (((任意字段=随机对照试验 OR 任意字段=随机) OR 任意字段=临床观察) OR 任意字段=疗效观察))](http://qikan.cqvip.com/Qikan/search/index?LngMySearHistoryIdGuid=b9d441d3-9c1c-467f-ad52-c6f2cacc8caf&from=Qikan_Article_History)
